# Three-dimensional transthoracic echocardiographic evaluation of tricuspid regurgitation severity using proximal isovelocity surface area: comparison with volumetric method

**DOI:** 10.1186/s12947-020-00225-y

**Published:** 2020-10-13

**Authors:** Beiqi Chen, Yu Liu, Wuxu Zuo, Quan Li, Dehong Kong, Cuizhen Pan, Lili Dong, Xianhong Shu, Junbo Ge

**Affiliations:** 1 Department of Echocardiography, Zhongshan Hospital, Fudan University, Shanghai, China; 2 Shanghai Institute of Medical Imaging, Shanghai, China; 3 Shanghai Institute of Cardiovascular Diseases, Shanghai, China

**Keywords:** Three-dimensional echocardiography, Tricuspid regurgitation, Proximal isovelocity surface area, Effective regurgitant orifice area

## Abstract

**Background:**

The quantification of tricuspid regurgitation(TR) using three-dimensional(3D) proximal isovelocity surface area (PISA) derived effective regurgitant orifice area (EROA) is feasible in functional TR. The aim of our study was to explore the diagnostic accuracy and utility of 3D PISA EROA in a larger population of different etiologies.

**Methods:**

One hundred and seven patients with confirmed TR underwent 2D and 3D transthoracic echocardiography (TTE). 3D PISA EROA was calculated and EROA derived from 3D regurgitant volume (Rvol) was used as the reference.

**Results:**

3D PISA EROA showed better correlation in primary TR than in functional TR(r = 0.897, *P* < 0.01). 3D PISA EROA differentiated severe TR with comparable accuracy in patients with primary and functional etiology (Z-value 16.506 vs 21.202), but with different cut-offs (0.49cm^2^ vs. 0.41 cm^2^). The chi-square value for incorporated clinical symptoms, positive echocardiographic results and 3D PISA EROA to grade severe TR was higher than only included clinical symptoms or incorporated clinical symptoms and positive echocardiographic results (chi-square value 137.233, *P* < 0.01).

**Conclusion:**

TR quantification using 3D PISA EROA is feasible and accurate under different etiologies. It has incremental diagnostic value for evaluating severe TR.

## Introduction

The unfavorable effect of TR has been gradually recognized [1–3]. Previous studies have shown that the mortality caused by moderate or above TR is twice as high as that of mild or below TR [4]. A retrospective study also found that the time interval for TR aggravation is related to the prognosis [5]. Therefore, quantification of TR consititues an important step for early treatment for these patients.

Echocardiography is the test of choice in diagnosing TR [6]. However, traditional quantitative methods such as vena contracta width(VCW) and two-dimensional (2D) proximal isovelocity surface area (PISA) method are insufficient to meet our needs [6]. The emergence of real-time three-dimensional(3D) echocardiography provides more possibilities in quantification of TR. Among them, effective regurgitant orifice area (EROA) has close relationship with hemodynamic consequences [6, 7]. Using the advantages of 3D imaging, it can also get rid of the geometric assumptions required by 2D PISA EROA and provide the real flow convergence [8].

Although EROA has been widely used and recommended in the assessment of mitral regurgitation(MR), due to the irregular shape of the tricuspid regurgitation orifice and the low flow velocity of TR compared to MR, the use of this method in quantitative TR is still to be studied [9, 10]. Previous studies have shown that 3D PISA EROA is more accurate than conventional 2D PISA EROA [8]. As a result, the 2D PISA EROA value of 0.4 cm^2^ for severe TR in the existing guidelines is not applicable to 3D PISA EROA [11–13]. In addition, other studies have found that the results of TR quantification using different methods for different etiologies are different [14]. Our study aims to explore the feasibility and accuracy of 3D PISA EROA in quantitative analysis of TR under different etiologies. The EROA derived from 3D regurgitant volume(Rvol) was used as the refrence method.

## Methods

### Population

From March 2019 to May 2020, we prospectively reviewed 107 patients at the Cardiac Surgery Department, Zhongshan Hospital who met the following inclusion criteria: (1) presence of TR verified by 2D transthoracic echocardiography(TTE) at the Department of Echocardiography in our hospital, (2) plan of isolated tricuspid valve surgery or mitral valve surgery combined with tricuspid valve surgery in a week, and (3) presence of a recognizable proximal flow convergence region of the tricuspid valve in the four-chamber view. The definition of TR was determined by echocardiographic findings, qualitative parameters and semiquantitative parameters. Exclusion criteria were: (1) absence of a recognizable proximal flow convergence region, (2) pulmonary regurgitation or pulmonary stenosis (mild and more), (3) intracardiac shunt, (4) presence of prosthetic tricuspid valve, (5) poor acoustic window, and (6) less than 18 years of age. According to the etiology, presence of atrial fibrillation and TR jet location based on 2D TTE, we divided patients into primary TR group and secondary TR group, with atrial fibrillation group and without atrial fibrillation group, and centric TR group and eccenric TR group [10]. Especially, the short-axis plane and the long-axis planes are used to determine the presence of organic tricuspid valve changes in primary TR. Organic tricuspid valve changes include rheumatic changes, congenital changes, trauma induced changes, endocarditis induced changes and degeneration. Secondary TR are determined with no structural abnormalities. It includes left-sided heart disease, atrial fibrillation, pulmonary arterial hypertension, right ventricle dysfunction and pace maker. All patients underwent echocardiography examination before the operation. The study was approved by the Medical Ethics Committee of Zhongshan Hospital Affiliated to Fudan University (Lot Number: B2018–117). All patients were enrolled after the signing of the informed consent.

### Two-dimensional TTE

**Fig. 1:**
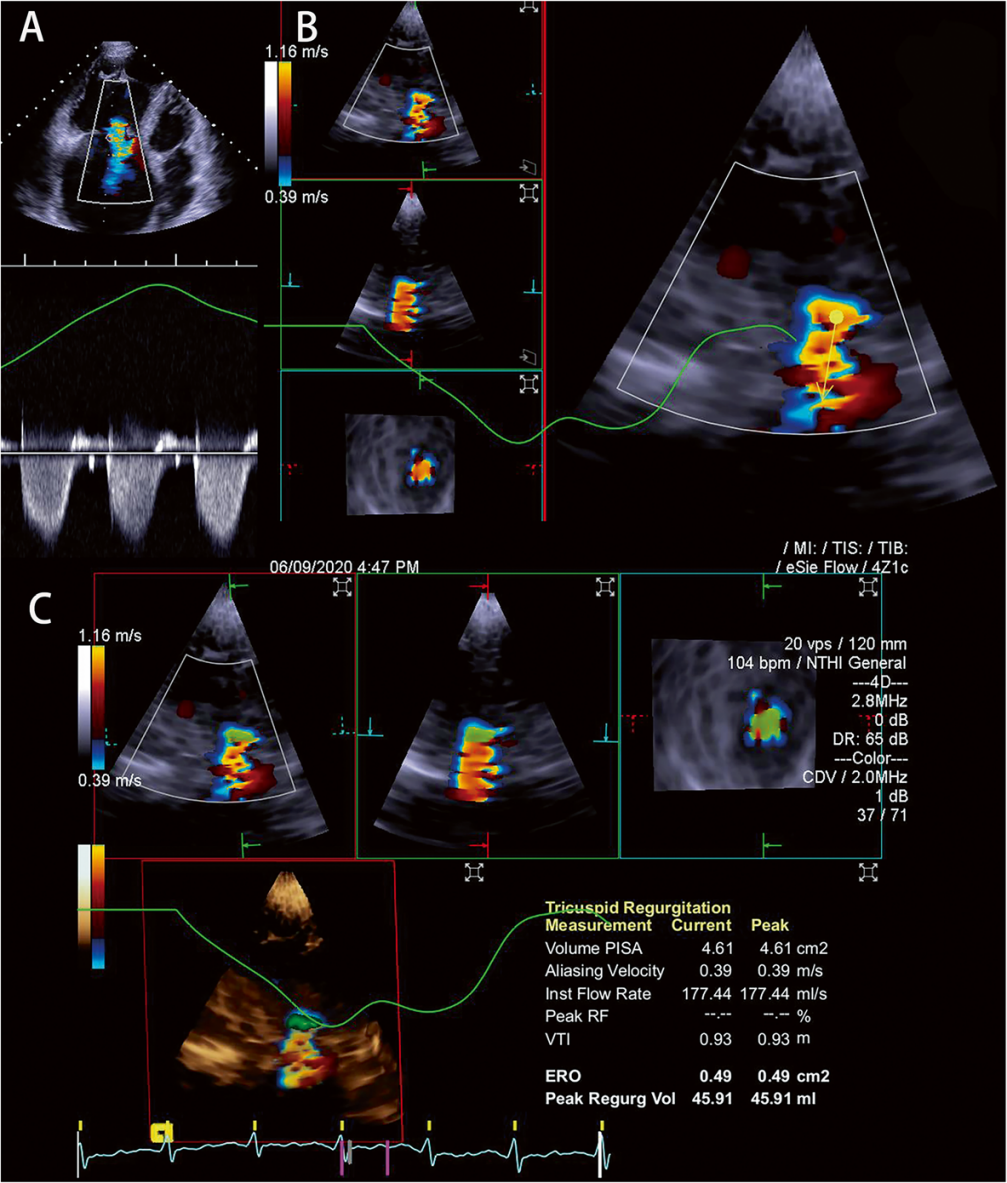
Measurement of 3D PISA EROA. **a** The TR VTI was measured by continuous-wave Doppler. **b** The arrow was set in direction of regurgitation. **c** The full volume ultrasound images were displayed in three orthogonal planes (left, four-chamber view; center, two-chamber view; right, short-axis view). PISA was visualized as green overlay on a 3D color Doppler image. 3D PISA EROA was calculated as (3D PISA x V_aliasing_)/ peak TR velocity

Each patient in the left lateral position underwent a standard 2D TTE examination [11, 15]. In patients with sinus rhythm, 3 consecutive cardiac cycle images were acquired; in patients with atrial fibrillation, atrial flutter, or pre-systolic systole, 6 consecutive cardiac cycle images were acquired [11, 15, 16]. Images were acquired using Siemens Acuson SC2000 Prime (Siemens Medical Solutions USA, Inc., Mountain View, CA) with 4v1c probe and were analyzed on its online workstation (Siemens Medical Solutions USA, Inc.). Velocity-time integral (VTI) of TR and maximum velocity(Vmax) of TR were determined by continuous-wave Doppler (Fig. 1a). The PISA was determined with color baseline shifting to 30.0 to 40.0 cm/sec and zoomed with the area of flow convergence. The hemispheric shape of flow convergence was chosen to measure the PISA radius in the frame with peak velocity during systole. 2D comprehensive multi-parameter method [6] was used to differentiate severe TR and non-severe TR.

### Three-dimensional TTE

Each patient in the left lateral position underwent a 3D TTE examination focusing on the tricuspid valve and the right ventricle(RV). In patients with sinus rhythm, 3 consecutive cardiac cycle images were acquired; in patients with atrial fibrillation, atrial flutter, or pre-systolic systole, 6 consecutive cardiac cycle images were acquired [11, 15, 16]. Images were collected using Siemens Acuson SC2000 Prime (Siemens Medical Solutions USA, Inc., Mountain View, CA) with 4Z1c probe and GE Vivid E95 (GE Vingmed Ultrasound, Horten Norway) with 4 V probe. Scanning depth, sector angle and line density were optimized to achieve frame rate of 20 to 25 frames/sec [17]. Multi-beat mode was attempted in patients with normal heart rhythm to improve spatial resolution.

**Fig. 2:**
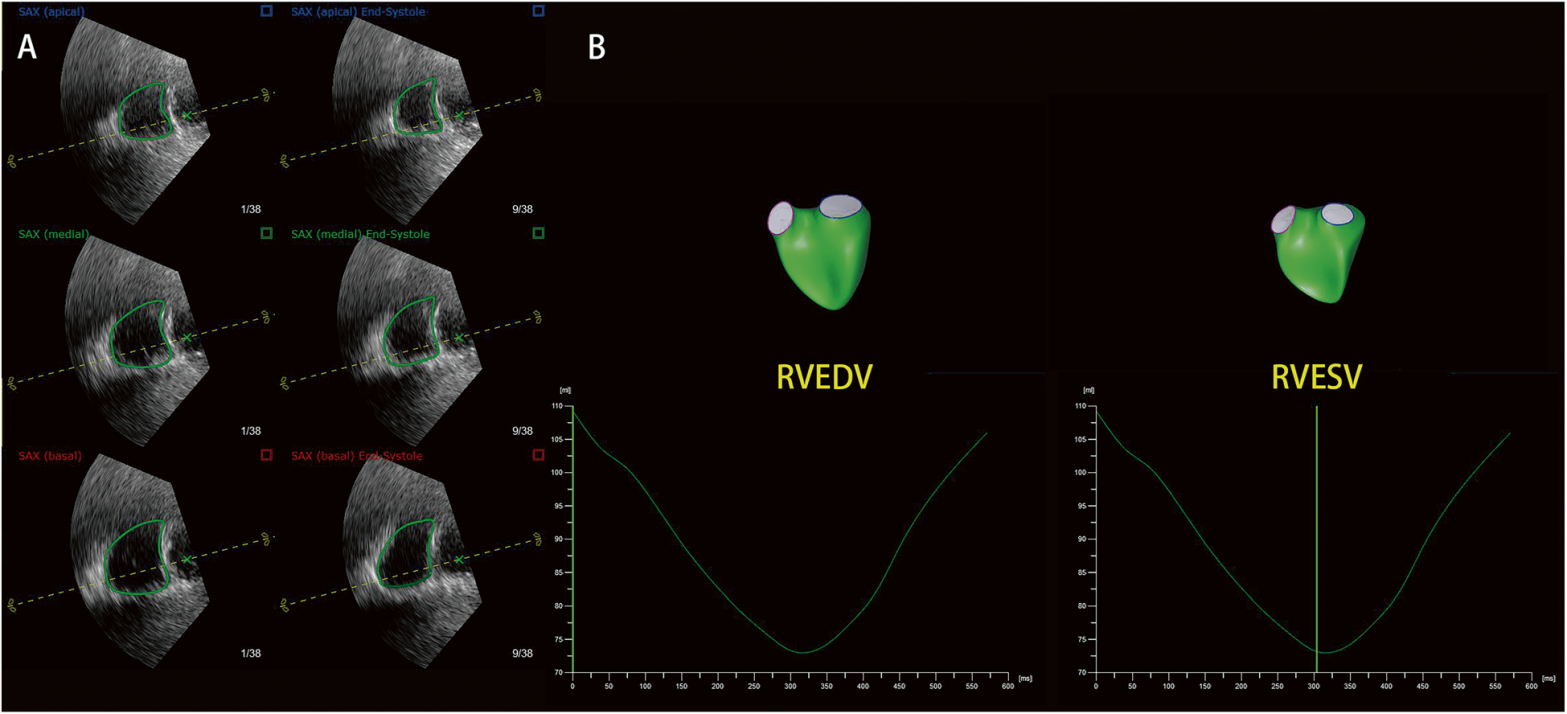
Measurement of 3D Rvol EROA. **a** RV was traced by dedicated software tools. **b** A pulsed-wave Doppler sample volume was placed into the RVOT to measure the VTI of outflow velocities. **c** Using the software to calculate RV volume from the 3D TTE data set, SV_RV_ was calculated by subtracting RVESV from RVEDV

On GE Vivid E95, a 3D full-volume data set of the entire RV and the tricuspid valve covering right ventricular outflow tract (RVOT) was acquired using 3D TTE. RV was traced in a 3D data set by dedicated software tools (Fig. 2a). The RVOT VTI was determined by pulse-wave Doppler at the position of the pulmonary valve annulus.

On Siemens Acuson SC2000 Prime, we selected the frame with peak velocity during systole, adopted eSie Flow preset and adjusted color baseline from 30.0 to 40.0 cm/sec. We set first point at the valve coaplation, drew arrow in direction of regurgitation and used zoom as needed (Fig. 1b).

### Imaging analysis

Images from Siemens Acuson SC2000 Prime were analyzed on-cart. Images from GE Vivid E95 were analyzed offline on TomTec (4D RV-Analysis; TomTec Imaging Systems, Unterschleissheim, Germany) workstation.

On TomTec, 2D PISA EROA was calculated using the following formula: 2*π* r^2^*V_aliasing_/Vmax (r is PISA radius, V_aliasing_ is the aliasing velocity of PISA, Vmax is TR Vmax) [18]. Stroke volume of RV (SV_RV_) was calculated by RV EDV subtracted RV ESV (RV EDV was the maximum of right ventricular volume, RV ESV was the minimum of right ventricular volume) (Fig. 2b) [19]. Stroke volume of RVOT (SV_RVOT_) was calculated by VTI_RVOT_ * S_RVOT_ (VTI_RVOT_ is RVOT VTI, S_RVOT_ is the area of RVOT calculating by measuring the diameter of the pulmonary annulus and using the circular area formula). Rvol is calculated by subtracting SV_RVOT_ from SV_RV_. The 3D Rvol EROA was calculated using the following formula: Rvol/VTI_TR_ (VTI_TR_ is TR VTI).

On Siemens Acuson SC2000 on-cart, Fig. 1c showed the full volume ultrasound images displayed in three orthogonal planes. The entire PISA of the TR was included in the volume data sets and visualized as a green overlay on the 3D color Doppler image (Fig. 1c). The 3D PISA EROA was calculated by the following formula: (3D PISA x V_aliasing_)/ peak TR velocity (V_aliasing_ is the aliasing velocity of 3D PISA) [20]. The final result was calculated automatically on its online workstation (Fig. 1c) [21].

### Statistical analysis

The data analysis was performed using SPSS 20.0 and MedCalc. The normality of continuous variables was tested by Kolmogorov-Smirnov test. Continuous variables were expressed as mean ± standard deviation. Categorical variables were expressed as percentages or frequency. Categorical variables were compared using Chi-square test. Continuous variables were compared using ANOVA test. Post hoc test of continuous variables adopted the Tukey test for normal distribution data and the Games-Howell test for non-normal distributions data, respectively. The correlation between 3D PISA EROA and 2D PISA EROA and the correlation between 3D PISA EROA and 3D Rvol EROA were tested using Spearman test. The differences between their correlation coefficients were tested using Wolfe Test. The data of 3D PISA EROA and 3D Rvol EROA first underwent the normality test again after logarithmic transformation, and then verified their consistency by Bland-Altman analysis [22]. Correlation between 3D PISA EROA and other indicators (etiology, sex, atrial fibrillation, TR jet location, VCW, PISA radius, TR VTI, TR Vmax, hepatic vein systolic reversal and dilated RV with preserved function) was tested using multivariate linear regression analysis. Taking 2D comprehensive multi-parameter method as reference [6], receiver-operator characteristic curve (ROC) analyses were used to access the ability of 3D PISA EROA to identify severe TR. The value closest to the upper left corner of the receiver operating characteristic curve was defined as Youden index or cut-off value, which had optimal sensitivity and specificity. The sequential logistic regression analysis was used to access the incremental diagnostic value of integrating multiple diagnostic indicators for diagnosing severe TR. 3D Rvol EROA≥0.4cm^2^ was used to define severe TR. Model 1 was clinical symptoms of right heart failure, such as lower limb edema, jugular vein distension and dyspnea. The positive echocardiographic results referring to the guideline [6] were forced into the Model 1 as Model 2, including abnormal/flail/large coaptation defect tricuspid valve, very large central jet or eccentric wall impinging jet, VCW ≥ 0.7 cm, PISA radius > 0.9 cm, systolic reversal of hepatic vein flow, RV with preserved function and 2D PISA EROA≥0.4cm^2^. As the Model 3, a 3D PISA EROA≥cut-off value was forced into the Model 2 to access the incremental diagnostic value. To examine the reproducibility of 3D PISA EROA measurements, the same observer and another independent blinded observer remeasured 3D PISA EROA of 20 randomly selected cases 1 month later, respectively. The reproducibility of 3D PISA EROA was tested using intraclass coefficient (ICC). All *P* values were tested by two-sided test, *P* < 0.05 was considered statistically significant.

## Results

### Baseline characteristics of the study population

A total of 107 patients were enrolled in our study, 8 were excluded because of incomplete images and 99 were finally included in the statistical analysis.

The distribution of etiology of TR was listed in Table 1. The basic information of 2D echocardiographic parameters were summarized in Table 2. There were no significant differences in the following indicators: sex, age, weight, height, BSA, atrial fibrillation, TR jet location, RVOT VTI, S_RVOT_ and SV_RVOT_. TR VTI and TR Vmax show significant differences (*P* < 0.001 and *P* = 0.013 respectively). As expected, the severe TR group had a greater number of following positive 2D echocardiographic findings (all *P* < 0.05): abnormal/flail/large coaptation defect tricuspid valve, VCW ≥ 0.7 cm, PISA radius > 0.9 cm, systolic reversal of hepatic vein flow and dilated RV with preserved function.

**Table 1:** Tricuspid valve pathogenic stratification based on two-dimensional transthoracic echocardiography. TR, tricuspid regurgitation

Variable	n (%)
Primary TR, overall	**44(44.4)**
Rheumatic changes	28(28.3)
Congenital changes	7(7.1)
Trauma	4(4.0)
Endocarditis	3(3.0)
Degeneration	2(2.0)
Secondary TR, overall	**55(55.6)**
Left-sided heart disease	37(39.4)
Atrial Fibrillation	8(8.1)
Pulmonary arterial hypertension	5(5.1)
Right ventricle dysfunction	3(3.0)
Pacemaker	2(2.0)

**Table 2:** Clinical and echocardiographic parameters of tricuspid regurgitation subgroups stratified by etiology (primary vs secondary) and severity

Variable	Primary TR		Secondary TR		P
	Non-severe (***n*** = 24)	Severe (***n*** = 20)	Non-severe (***n*** = 28)	Severe (***n*** = 27)	
Sex,Female(%)	15(62.5)	10(50.0)	18(64.3)	18(66.7)	0.677
Age(y)	54.5 ± 11.6	56.3 ± 13.1	66.4 ± 7.5^*^	61.9 ± 9.6	0.010
Weight(kg)	58.2 ± 8.3	61.2 ± 12.6	59.8 ± 11.1	63.0 ± 21.2	0.729
Height(cm)	162.3 ± 6.7	164.2 ± 8.9	162.1 ± 9.1	156.0 ± 25.2	0.319
BSA(m^2^)	1.6 ± 0.1	1.6 ± 0.2	1.6 ± 0.2	1.6 ± 0.2	0.702
Atrial Fibrillation(%)	12(50.0)	7(35.0)	9(32.1)	12(44.4)	0.544
TR jet location, centric(%)	6(25.0)	6(30.0)	15(53.6)	18(66.7)	0.417
Malcoaptation or flail leaflet(%)	10(41.7)	15(75.0)	3(10.7)	10(37.0)	< 0.001
VCW,≥0.7 cm(%)	4(16.7)	19(95.0)	3(10.7)	23(85.2)	< 0.001
PISA radius, > 0.9 cm(%)	0(0)	4(20.0)	0(0)	1(3.7)	0.007
TR VTI(m)	0.73 ± 0.20	0.61 ± 0.21	0.89 ± 0.21^+^	0.67 ± 0.20	< 0.001
TR Vmax(m/s)	2.40 ± 0.56	2.17 ± 0.65	2.69 ± 0.62^+^	2.23 ± 0.60	0.013
RVOT VTI(m)	0.12 ± 0.02	0.12 ± 0.03	0.15 ± 0.05	0.14 ± 0.05	0.350
S(RVOT)(cm^2^)	3.16 ± 0.60	3.93 ± 1.61	3.48 ± 0.87	3.28 ± 0.87	0.237
SV(RVOT)(ml)	38.8 ± 9.3	46.7 ± 16.0	50.3 ± 22.0	42.4 ± 10.9	0.207
RVEDV(ml)	107.9 ± 21.8^+^	173.4 ± 46.6	129.2 ± 34.9^*,+^	162.8 ± 43.5	< 0.001
RVESV(ml)	44.9 ± 13.5	64.9 ± 26.9	57.5 ± 28.6	65.6 ± 20.7	0.088
SV(RV)(ml)	63.0 ± 12.8^+^	107.5 ± 29.6	70.4 ± 21.1^+^	97.4 ± 36.9	< 0.001
Rvol(ml)	24.2 ± 12.5^+^	60.9 ± 23.1	20.8 ± 10.8^+^	53.8 ± 31.4	< 0.001
Hepatic vein systolic reversal(%)	2(8.3)	14(70.0)	2(7.1)	15(55.6)	< 0.001
Dilated right ventricle with preserved function(%)	8(33.3)	15(75.0%)	7(25.0)	23(85.2)	< 0.001
2D PISA EROA(cm^2^)	0.12 ± 0.02^+^	0.85 ± 0.38	0.12 ± 0.02^+^	0.57 ± 0.10	0.008
3D PISA EROA(cm^2^)	0.26 ± 0.04^+^	1.10 ± 0.46	0.23 ± 0.03^*,+^	0.92 ± 0.17	< 0.001
3D Rvol EROA(cm^2^)	0.39 ± 0.04^+^	1.27 ± 0.28	0.23 ± 0.03^+^	0.83 ± 0.13	0.015

*TR* tricuspid regurgitation, *VCW* vena contrata width, *PISA* proximal isovelocity surface area, *VTI* velocity-time integral, *Vmax* maximum velocity, *RVOT* right ventricular outflow tract, *S* area, *SV* stroke volume, *RVEDV* maximum of right ventricular volume, *RVESV* minimum of right ventricular volume, *RV* right ventricle, *Rvol* regurgitant volume, *2D* two-dimensional, *3D* three-dimensional, *EROA* effective regurgitant orifice area

*P < 0.05 vs. Primary TR. +P < 0.05 vs. Severe TR

The 3D echocardiographic parameters were summarized in Table 2. There were significant differences in all 3D echocardiographic variables (all P < 0.05), except for RV ESV. RV EDV, SV_RV_ and Rvol were significantly larger in severe TR patients.

### TR quantification by the 3D PISA EROA compared with 2D PISA EROA and 3D Rvol EROA

**Fig. 3:**
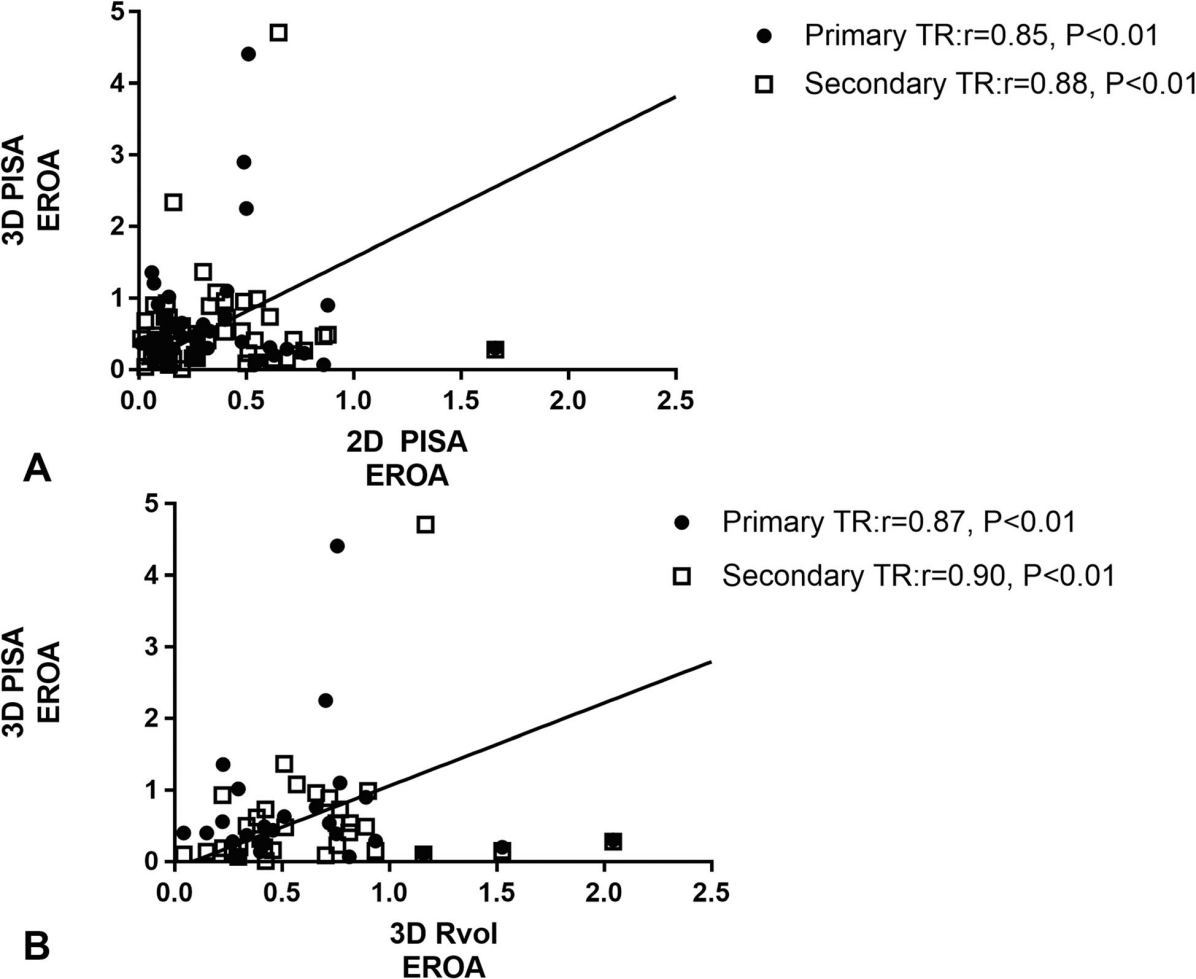
Regression plots showing correlations of 3D PISA EROA with 2D PISA EROA (**a**) and 3D Rvol EROA (**b**)

The results of three quantitative methods: 2D PISA EROA, 3D PISA EROA and 3D Rvol EROA were reported in Table 2. The results of the Spearman correlation coefficients were reported in Table 3. Correlations between 3D PISA EROA and 2D PISA EROA and that between 3D PISA EROA and 3D Rvol EROA in the subgroups of etiologies were showed in Fig. 3a and b, respectively.

**Table 3:** Spearman correlation coefficients of 2D PISA EROA, 3D Rvol EROA and 3D PISA EROA

Variable	2D PISA EROA vs 3D PISA EROA		3D PISA EROA vs 3D Rvol EROA	
	r	P	r	P
Total patient population	0.878	< 0.01	0.884	< 0.01
Primary TR	0.848	< 0.01	0.866	< 0.01
Secondary TR	0.88	< 0.01	0.897	< 0.01
With atrial fibrillation	0.721	< 0.01	0.59	< 0.01
Without atrial fibrillation	0.97	< 0.01	0.97	< 0.01
Eccentric TR	0.784	< 0.01	0.692	< 0.01
Centric TR	0.97	< 0.01	0.973	< 0.01

*TR* tricuspid regurgitation, *2D* two-dimensional, *3D* three dimensional, *Rvol* regurgitant volume, *PISA* proximal isovelocity surface area, *EROA* effective regurgitant orifice area

**Fig. 4:**
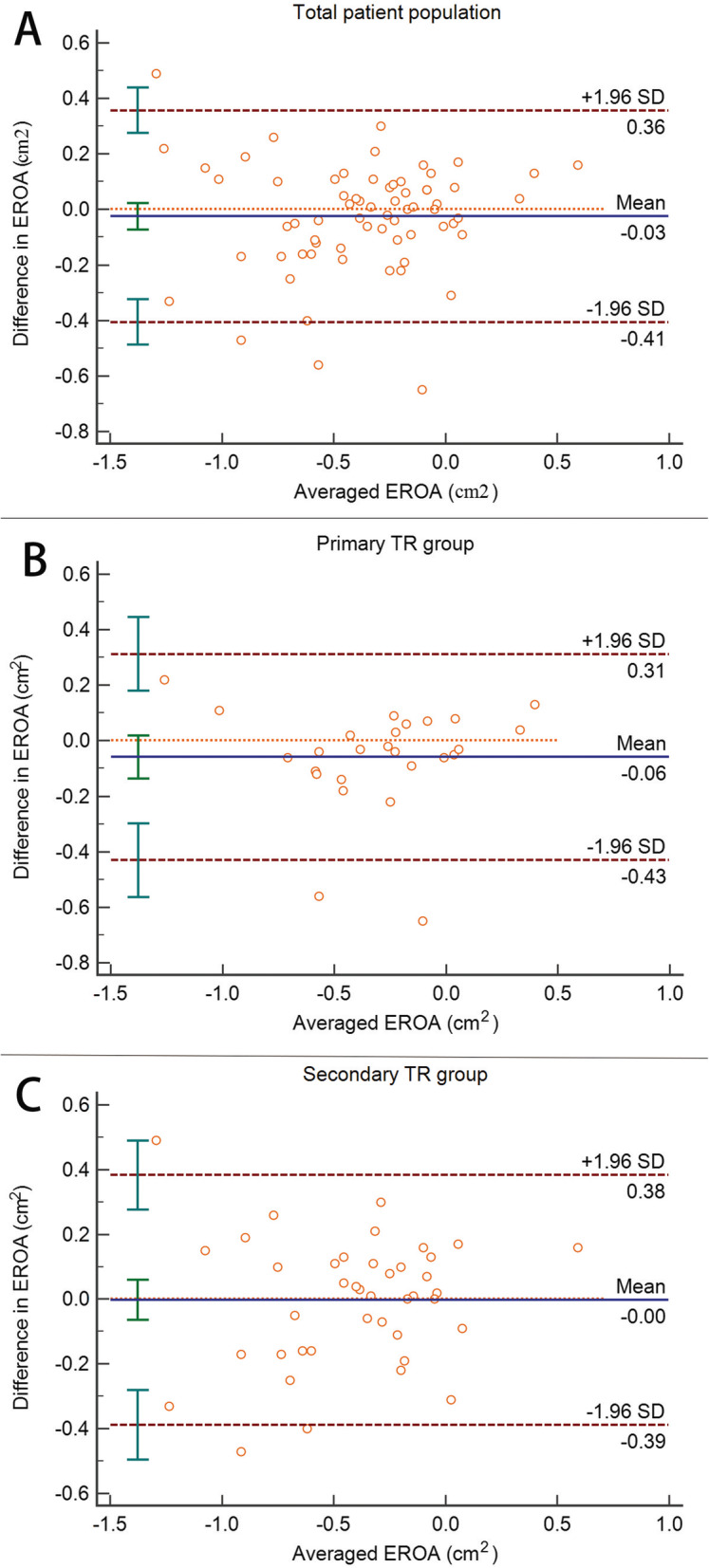
Bland-Altman plots showing unacceptable agreement but negligible systematic bias between 3D PISA EROA and 3D Rvol EROA PISA in total patient population (**a**), primary TR group (**b**) and secondary TR group (**c**)

In the total patient population, 3D PISA EROA correlated well with both 2D PISA EROA and 3D Rvol EROA (*P* = 0.85 in Wolfe Test). Irrespective of methods, the correlations demonstrated both high agreement in primary TR and secondary TR (*P* = 0.54 in Wolfe Test when comparing 3D PISA EROA with 2D PISA EROA; *P* = 0.50 in Wolfe Test when comparing 3D PISA EROA with 3D Rvol EROA). Although Bland-Altman analysis showed unacceptable agreement between 3D PISA EROA and 3D Rvol EROA in the total patient population and the subgroups of etiology, the systematic bias was negligible (7% underestimation in the total patient population, 15% underestimation in the primary TR group and no bias in the secondary TR group) (Fig. 4a, b and c).

As expected, irrespective of methods, the atrial fibrillation group showed significantly weaker correlation than no atrial fibrillation group and the eccentric TR group showed significantly weaker correlation than the centric TR group, too.

### Diagnostic value of 3D PISA EROA to differentiate non-severe TR and severe TR

**Fig. 5:**
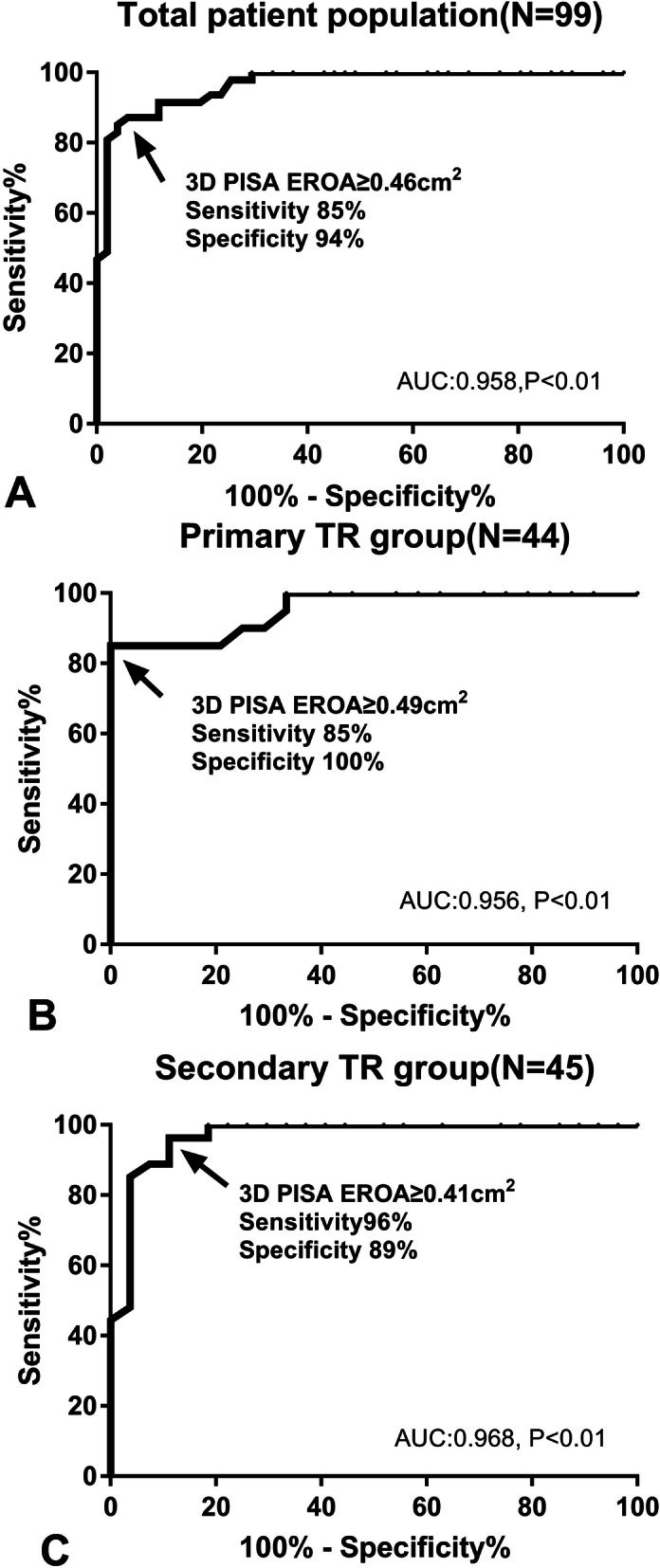
**a** ROC analysis shows 3D PISA EROA for a cut-off value of 0.46 cm^2^ with high diagnostic accuracy in the total patient population. **b**, **c** In the subgroup with primary TR and secondary TR, ROC analyses also show each 3D PISA EROA cut-off value for severe TR (0.49 cm^2^ and 0.41cm^2^, respectively) with high diagnostic accuracy. AUC, Area under the curve

In the total patient population, as accessed by ROC analysis, the optimal cut-off value of 3D PISA EROA was 0.46cm^2^ with sensitivity of 85% and specificity of 94% (Fig. 5a. The AUC was 0.958 to differentiate non-severe TR and severe TR (Z = 26.11, *P* < 0.01). However, the optimal cut-off values of 3D PISA EROA in primary TR group and secondary TR group were different. The primary TR group showed the cut-off value of 0.49cm^2^ (sensitivity of 85%, specificity of 100%) with the AUC of 0.956 (Z = 16.51, P < 0.01) (Fig. 5b). The secondary TR group showed the cut-off value of 0.41cm^2^ (sensitivity of 96%, specificity of 89%) with the AUC of 0.968 (Z = 21.20, P < 0.01) (Fig. 5c).

### Incremental value of 3D PISA EROA for integrated approach to grade TR severity

Multivariate linear regression analysis on 3D PISA EROA (R^2^ = 0.614) showed that 3D PISA EROA was affected by several 2D echocardiographic indicators (Table 4). As seen in Table 4, VCW, PISA radius, TR Vmax and hepatic vein systolic reversal have the significant impact on 3D PISA EROA.

**Table 4:** Multivariate linear regression analysis results of 3D PISA EROA

Variable	B	Std. Error	Beta	t	P	VIF
Etiology(primary/ secondary)	−0.162	0.089	−0.127	−1.826	0.071	1.103
Sex(male/female)	−0.111	0.092	−0.085	−1.207	0.231	1.135
Atrial fibrillation (with/without)	−0.002	0.092	−0.001	− 0.018	0.986	1.151
TR jet location (eccentric/centric)	0.014	0.095	0.010	0.144	0.885	1.155
VCW	−0.291	0.104	−0.230	−2.802	< 0.01	1.535
PISA radius	−1.162	0.210	−0.402	−5.539	< 0.01	1.199
TR VTI	−0.467	0.357	0.179	1.308	0.194	4.286
TR Vmax	−0.464	0.133	−0.460	−3.477	< 0.01	3.981
Hepatic vein systolic reversal (with/ Without)	−0.308	0.107	−0.229	−2.862	< 0.01	1.459
Dilated right ventricle with preserved function (with/without)	0.011	0.099	0.009	0.110	0.913	1.384

*TR* tricuspid regurgitation, *VCW* vena contracta width, *PISA* proximal isovelocity surface area, *VTI* velocity-time integral, *Vmax* maximum velocity

**Fig. 6:**
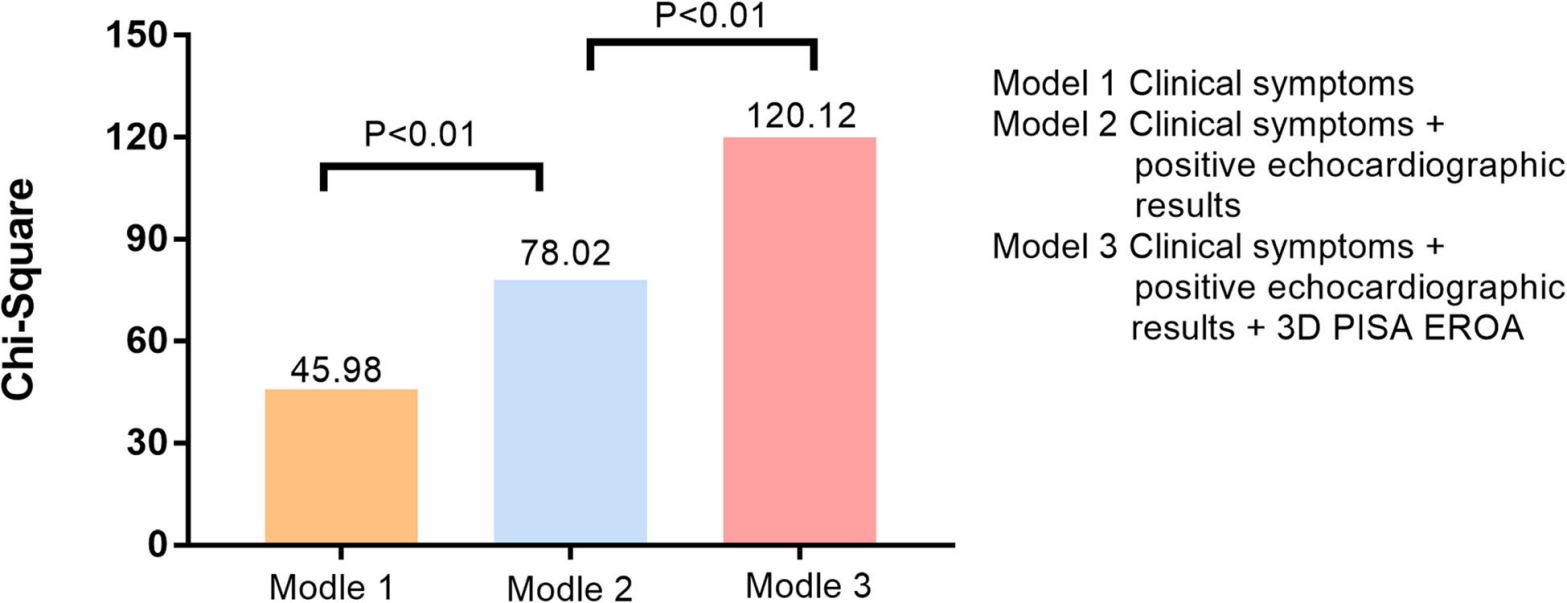
Incremental utility of 3D PISA EROA to evaluate 3D RVol EROA-based severe TR

In Fig. 6, the Chi-square value for that incorporating clinical symptoms, positive echocardiographic results and positive 3D PISA EROA values (primary TR: 3D PISA EROA ≥0.49cm^2^, secondary TR: 3D PISA EROA≥0.41cm^2^) to evaluate severe TR is higher than that only based on the clinical symptoms and combining clinical symptoms and positive echocardiographic results (120.1 vs 78.0 and 46.0).

### Reproducibility of 3D VC assessment

The intra-observer ICC was 0.943 (95% CI: 0.919–0.987), and the inter-observer ICC was 0.843 (95% CI: 0.728–0.957). There was good intra-observer consistency as well as good inter-observer consistency for 3D PISA EROA.

## Discussion

Our study demonstrates the following points for the first time: (1) 3D PISA EROA correlates well with 3D Rvol EROA in different etiological subgroups of TR; (2) the optimal cut-off values differ, being higher in primary TR; and (3) 3D PISA EROA has incremental diagnostic value over clinical symptoms and positive echocardiographic results for the integrated approach in grading TR severity.

### Comparison between 2D PISA methods and 3D PISA EROA

The low-speed characteristics and irregular regurgitant orifice of TR deviate from the hypothetical model of the PISA method, leading to a possibility of underestimation theoretically [9]. And previous study has verified it [14, 23, 24]. The EROA by PISA method quantifies the severity of regurgitation based on one flame with peak regurgitant velocity, leading to a possibility of overestimation in measuring EROA [25, 26]. Among them, 3D PISA EROA overcomes the geometric assumption, being more accurate [8]. This may account for the results that in our study, PISA radius of > 0.9 cm is seen in only about 10% of severe TR group and the mean EROA by 2D is lower than 3D PISA EROA.

### Comparison between 3D PISA EROA and 3D Rvol EROA

As expected, 3D PISA EROA can be affected by atrial fibrillation and TR jet location. In TR with atrial fibrillation and eccentric jet, 3D PISA EROA correlates weak with both 2D PISA EROA and 3D Rvol EROA.

3D PISA EROA is measured on the frame of peak velocity during systole. However, the EROA is dynamically changing and the single-frame snapshot of it may not represent the regurgitation severity of the entire systole [25, 26]. Previous study verified that the frame of highest regurgitant velocity could be used to grade the severity of mitral regurgitation [26, 27]. In practical use, the selection of frame usually depends on the peak of the CW spectrum for measurement. However, unlike mitral regurgitation, TR’s regurgitant flow velocity is lower and its regurgitant orifice is larger. The CW spectrum of TR is not as concentrated as that of mitral regurgitation. The CW spectrum of TR tends to present as blurred peak boundaries, leading to deviations when selecting the frame with the highest regurgitant flow velocity [8]. Among patients with atrial fibrillation, variability of regurgitant severity may be more remarkable owing to different hemodynamic condition. When it comes to TR jet location, 3D PISA EROA overcomes the geometric assumption of PISA [28–30], however, still presents dissatisfied results in eccentric TR. This is because the septal leaflet is the shortest one with the smallest range of motion [31]. In most cases, TR occurs along the septal leaflet [31, 32] and renders it difficult to put the CW sampling line parallel to TR jet in four-chamber view.

Previous study showed that the correlation between 3D vena contracta area(VCA) and 2D PISA-EROA demonstrated significantly better agreement in patients with primary TR than secondary TR [14]. de Agustin’s study showed good correlation between 3D PISA EROA and both 2D PISA EROA and 3D Rvol EROA among secondary TR patients, being better between 3D PISA EROA and 3D Rvol EROA [8]. Our study demonstrates good correlations between 3D PISA EROA and both 2D PISA EROA and 3D Rvol EROA, which exists not only in secondary TR but also primary TR. Wolfe Test showed two similar correlation coefficients. Patients with larger atrial fibrillation population compared to de Agustin’s study may account for the difference that similar correlations between 3D PISA EROA and both 2D PISA EROA and 3D Rvol EROA instead of better correlation between 3D PISA EROA and 3D Rvol EROA exist. In our study, the mean 3D PISA EROA values are much closer to 3D Rvol EROA than 2D PISA EROA, while the correlation coefficients are about the same. Correlation coefficient reflects the deviation of two variables from their respective mean values rather than the systematic difference between these variables, which could be characterized by the differences between mean values. As a result, variables can be highly correlated but with systemic bias. As in our study, the similar correlation coefficient but different means between 3D PISA EROA and both 2D PISA EROA and 3D Rvol EROA should be ascribed to systemic deviation.

As 2D PISA EROA is relatively inaccurate in quantification of TR owing to its hemispheric assumption [8, 9, 28–30], 3D Rvol EROA, as an indicator that is free from geometric assumption is supposed to show better correlation with 3D PISA EROA. However, their correlation coefficients are similarly high in our study. This might be explained by the similar single-frame method between 2D PISA EROA and 3D PISA EROA which compensates the error of geometric assumption. 3D PISA EROA and 3D Rvol EROA, in contrast, are both free from the error of geometric assumption but their different timing of calculation may have caused their systematic difference.

### An integrated method for grading TR severity

Our study finds that VCW, PISA radius, TR Vmax and hepatic vein systolic reversal are all factors that can affect 3D PISA EROA. 3D PISA EROA has an added diagnostic value for severe TR on the basis of clinical symptoms and positive 2D echocardiographic results. The 3D PISA EROA value of severe TR obtained in our study has high sensitivity and high specificity. In the sequential logistic regression analysis, combining clinical symptoms, positive 2D echocardiographic results and 3D PISA EROA has the highest chi-square value. As a result, the incremental diagnostic value of 3D PISA EROA may be considered in treatment selection for TR in the future.

### Limitation

Our study didn’t engage cardiac magnetic resonance as the reference for volume measurement. Previous study showed that 3D echocardiography underestimated the volume of the RV [33]. However, when the RVEDV is less than 300 ml, the volume measured by 3D echocardiography has good consistency with cardiac magnetic resonance, and the underestimation at this time can be ignored [33]. In the current study, all patients referred to our center had guideline directed medical therapy at presence, and the RVEDV of patients included in the statistics were all less than 300 ml, which would be less affected by the systematic bias of 3D echocardiography.

The handling of papillary muscles in RV volume calculation may also cause over- or underestimation of the regurgitant volume. The SV_RVOT_ calculated was based on the diameter of RVOT from a single 2D view, which may be different from the actual shape of RVOT [34].

3D PISA EROA is still a method based on Doppler technology, resulting in its angle dependency. The surface it displays may still not be a true surface of isovelocity, but a surface of iso-Doppler velocity [35].

When the images obtained are not optimal, the utility of 3D transthoracic echocardiography is limited, especially affecting atrial fibrillation patients and eccentric TR patients. Cardiac magnetic resonance will be more ideal under these circumstances.

## Conclusion

3D PISA EROA is a reliable method in quantification of TR. The performance of 3D PISA EROA in primary TR and secondary TR differs and based on the difference, the diagnostic value of grading severe TR is high. These results may help in treatment selection for TR in the future.

## Data Availability

The datasets used and/or analyzed during the current study are available from the corresponding author on reasonable request.
